# Supplementary material for: Physico-Chemical Characterization and Antimicrobial Properties of Hybrid Film Based on Saponite and Phloxine B
Source: Molecules. 2021 Jan 10;26(2):325. doi: 10.3390/molecules26020325 (PMC7827291; doi:10.3390/molecules26020325)
Supplement: Supplementary file 1 [file molecules-26-00325-s001.pdf]

Supplementary data

Physico-chemical characterization and antimicrobial properties of hybrid film based on saponite and phloxine B.

Nitin Chandra Teja Dadi[1], Matúš Dohál[1], Veronika Medvecká[2] Juraj Bujdák[3,4], Kamila Koči[1], Anna Zahoranová[2], Helena Bujdáková[1*]

[1]*Comenius University in Bratislava, Faculty of Natural Sciences, Department of Microbiology and Virology, Bratislava, Slovak Republic*

[2]*Comenius University in Bratislava, Faculty of Mathematics, Physics and Informatics, Department of Experimental Physics, Bratislava, Slovak Republic*

[3]*Comenius University in Bratislava, Faculty of Natural Sciences, Department of Physical and Theoretical Chemistry, Bratislava, Slovak Republic*

[4]*Department of Inorganic Chemistry of SAS, Bratislava, Slovak Republic*

Corresponding author

prof. Helena Bujdakova, PhD.

Comenius University in Bratislava, Faculty of Natural Sciences

Department of Microbiology and Virology

Ilkovičova 6

842 15 Bratislava

Slovak Republic

helena.bujdakova@uniba.sk

Detection of the *MecA* gene of *S. aureus*

The the *MecA* gene was detected with primers synthesized by Metabion International AG, Germany, which had the following sequences: Forward: GTA GAA ATG ACT GAA CGT CCG ATA A, Reverse: CCA ATT CCA CAT TGT TTC GGT CTA A (Braoios et al., 2009). The final volume of the reaction mixture for the PCR reaction was 20 µl and was prepared with the reagents given in Table S1. In this the genomic DNA of the 3953, L 12 and L 18 strains were used as templates. The PCR cycle described in Table S2 was performed in a thermocycler (C1000, BIO-RAD, USA). PCR products were visualized by electrophoresis in a 1% agarose gel at 90 V, 90 min (Power Pac, BIO-RAD, USA) with GoodView Nucleic Acid Stain-HGV-II in a UV transilluminator (MUV-21-312-220, Major Science, USA).

Table S1: Reaction mixture for PCR assay of the *MecA* gene.

**Reagents**	**Quantity**
5x HOT FIREPol Blend Master Mix	4 µL
DNA template	1 µL
Forward primer	0,5 µL
Reverse primer	0,5 µL
DNA free water	14 µL

Table S2: PCR cycle for the *MecA* gene.

**Steps**	**Temperature (°C)**	**Time (min.)**	**Cycles**
Initial denaturation	95	15:00	1x
Denaturation	95	00:20	34x
Annealing	51	01:00	
Extension	72	01:00	
Final extension	72	10:00	1x

To determine the resistance of *S. aureus* strains 3953, L12, and L18 to methicillin, the presence of the *MecA* gene was identified by PCR analysis followed by agarose gel electrophoresis. A clear signal was visualized at 310 bp in clinical isolates L12 and L18, confirming the MRSA phenotype. On the other hand, no signal was identified in the standard strain 3953 (Figure S1).

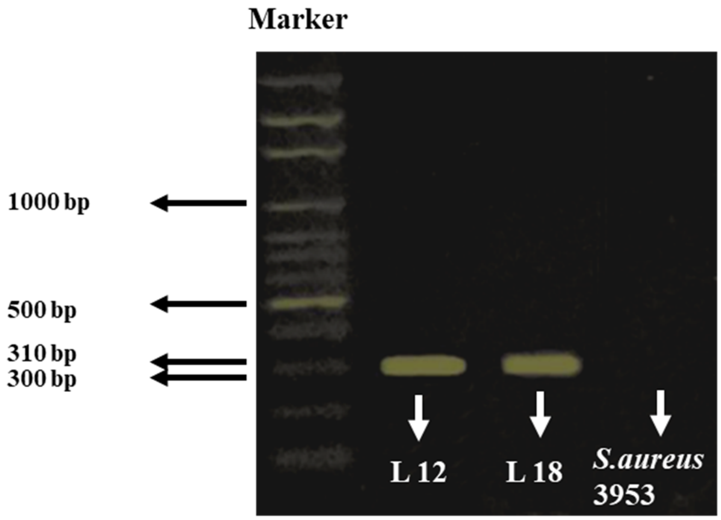

Figure S1: Agarose gel electrophoresis showing amplicons of the *MecA* gene in *S. aureus* resistant strains of L18 and L12. The *MecA* gene can be viewed at 310 bp. No visible band was observed in sensitive *S. aureus* 3953.
